# The Role of ArtificiaI Intelligence in Brain Tumor Diagnosis: An Evaluation of a Machine Learning Model

**DOI:** 10.7759/cureus.61483

**Published:** 2024-06-01

**Authors:** Adriel Abraham, Rejath Jose, Nabeel Farooqui, Jonathan Mayer, Jawad Ahmad, Zain Satti, Thomas J Jacob, Faiz Syed, Milan Toma

**Affiliations:** 1 Department of Internal Medicine, New York Institute of Technology College of Osteopathic Medicine, New York, USA; 2 Department of Computer and Information Science, University of Pennsylvania School of Engineering and Applied Science, Philadelphia, USA; 3 Department of Clinical Sciences, New York Institute of Technology College of Osteopathic Medicine, New York, USA; 4 Department of Osteopathic Manipulative Medicine, New York Institute of Technology College of Osteopathic Medicine, New York, USA

**Keywords:** medical diagnosis, pituitary tumors, meningiomas, gliomas, f1 score, precision and recall, google teachable machine, mri imaging, brain tumor classification, machine learning

## Abstract

This research study explores of the effectiveness of a machine learning image classification model in the accurate identification of various types of brain tumors. The types of tumors under consideration in this study are gliomas, meningiomas, and pituitary tumors. These are some of the most common types of brain tumors and pose significant challenges in terms of accurate diagnosis and treatment.

The machine learning model that is the focus of this study is built on the Google Teachable Machine platform (Alphabet Inc., Mountain View, CA). The Google Teachable Machine is a machine learning image classification platform that is built from Tensorflow, a popular open-source platform for machine learning. The Google Teachable Machine model was specifically evaluated for its ability to differentiate between normal brains and the aforementioned types of tumors in MRI images.

MRI images are a common tool in the diagnosis of brain tumors, but the challenge lies in the accurate classification of the tumors. This is where the machine learning model comes into play. The model is trained to recognize patterns in the MRI images that correspond to the different types of tumors. The performance of the machine learning model was assessed using several metrics. These include precision, recall, and F1 score. These metrics were generated from a confusion matrix analysis and performance graphs. A confusion matrix is a table that is often used to describe the performance of a classification model. Precision is a measure of the model's ability to correctly identify positive instances among all instances it identified as positive. Recall, on the other hand, measures the model's ability to correctly identify positive instances among all actual positive instances. The F1 score is a measure that combines precision and recall providing a single metric for model performance. The results of the study were promising.

The Google Teachable Machine model demonstrated high performance, with accuracy, precision, recall, and F1 scores ranging between 0.84 and 1.00. This suggests that the model is highly effective in accurately classifying the different types of brain tumors. This study provides insights into the potential of machine learning models in the accurate classification of brain tumors. The findings of this study lay the groundwork for further research in this area and have implications for the diagnosis and treatment of brain tumors. The study also highlights the potential of machine learning in enhancing the field of medical imaging and diagnosis. With the increasing complexity and volume of medical data, machine learning models like the one evaluated in this study could play a crucial role in improving the accuracy and efficiency of diagnoses. Furthermore, the study underscores the importance of continued research and development in this field to further refine these models and overcome any potential limitations or challenges. Overall, the study contributes to the field of medical imaging and machine learning and sets the stage for future research and advancements in this area.

## Introduction

Brain tumors are abnormal growths of cells within the skull, which can be either primary, originating from brain cells, or secondary, resulting from cancer spread (metastasis) from other body parts. The early detection of brain tumors is crucial for improving patient outcomes, as it can significantly reduce morbidity and mortality rates [1]. Among the various types of brain tumors, gliomas, meningiomas, and pituitary tumors are particularly notable due to their prevalence and the challenges they present in diagnosis and treatment. Current diagnoses of brain tumors involve the use of different imaging modalities, including magnetic resonance imaging (MRI) and computed tomography (CT) scans. MRI provides key information on the anatomical structure of human tissue via soft tissue contrast, making it essential for the identification of the brain tumor’s size, shape, and location [2].

However, the challenge lies in the ability of MRIs to properly classify tumors. Conventional MRI classification and grading of gliomas, a specific type of brain tumor, ranges from 55.1% to 88.3% in accuracy and has even demonstrated a 50% false-positive rate [3]. This holds tremendous clinical significance as early accurate detection of brain tumors is associated with improved treatment and survival [4]. The reason behind this is its difficulty in segmentation [5,6]. MRIs segment a tumor in several visualized images in order to view multiple features of the tumor. Some tumors, such as meningiomas, are easily segmented; however, gliomas are difficult to localize and are often diffuse and poorly contrasted, thus posing a challenge in segmentation.

The modeling of the segments into data-based models also plays a role in the difficulty of proper tumor classification. The 3D data-based model provides more contextual information; however, they require manual segmentation thus taking significantly more time when scanning, and are still susceptible to inaccuracies. The incorporation of machine learning could pose a possible solution. Machine learning uses neural networks from minimal inputted data to solve scenarios and is beneficial in medical imaging via pattern recognition and identification [7]. The model in this study aims to combat the aforementioned issues as it utilizes machine learning to accurately classify several brain tumors, therefore improving the number of missed diagnoses of brain tumors.

Several advanced MRI classification models, such as dynamic contrast-enhanced (DCE) and magnetic resonance spectroscopy (MRS), are in use today to improve the precision of tumor detection [8,9]. DCE allows for the quantitative evaluation of intravascular contrast diffusion within the interstitium, a frequent phenomenon in brain tumors. However, the intricacies of its acquisition and analysis methods restrict its clinical application and accessibility [10]. MRS, which incorporates molecular abnormalities, provides valuable insights into brain tumors. Yet, its clinical application is rare due to its lengthy process, variability across different imaging locations, and the necessity for a technologist or radiologist's assistance [11]. 

Recent advancements in machine learning have led to the development of sophisticated image classification models that can accurately identify different types of brain tumors. Convolutional neural networks (CNNs) are at the forefront of these developments, with architectures designed to process and analyze MRI images for tumor detection [12-14]. A CNN model with four convolutional layers, ReLU activation functions, dropout layers, and max-pooling layers was proposed, achieving an accuracy of 97.39% and an average F1-Score of 96.11% in one test [12]. The model used a 10-fold cross-validation method, which is a technique to evaluate the model's performance by partitioning the data into subsets and using each in turn for testing. The image input size for the CNN was set to 256 × 256 pixels, and the classification output was divided into three classes corresponding to meningioma, glioma, and pituitary tumor.

In addition to CNNs, other deep-learning methods and machine-learning techniques have been explored for brain tumor detection. For instance, a study proposed two deep learning methods and several machine learning approaches, achieving training accuracies of 96.47% and 95.63% for the 2D CNN and auto-encoder network, respectively [1]. The areas under the ROC curve for both networks were impressively high, at 0.99 or 1, indicating excellent classification performance. Transfer learning has also been employed to enhance classification accuracy. By using pre-trained models such as EfficientNets and MobileNetv3, researchers have been able to achieve significant performance improvements. For example, EfficientNetB2 yielded an overall test accuracy of 99.06%, while MobileNetv3 achieved the highest accuracy of 99.75% [13,14]. Transfer learning is particularly beneficial when dealing with limited labeled medical data, as it allows the use of knowledge acquired from extensive benchmark datasets like ImageNet [13,14].

Hybrid methods that combine CNNs with other algorithms like support vector machines (SVM) or artificial neural networks (ANN) have been developed to extract deep feature maps with high accuracy [15]. These hybrid models utilize both deep features from CNNs and handcrafted features to produce highly efficient features for distinguishing between types of brain tumors [15]. Comparative studies have shown that different architectures can yield varying levels of accuracy, sensitivity, specificity, and F1 scores. For instance, AlexNet, VGG16, and ResNet-50 have shown accuracies ranging from 95.60% to 97.66%, with a hybrid model of VGG16 and ResNet 50 reaching an accuracy of nearly 100% [16].

This study examines the application of machine learning, specifically image classification models, in the accurate identification of these brain tumor types using MRI images [12,16]. Specifically, the model under consideration in this study addresses the aforementioned challenges by requiring only internet access, thereby providing wide accessibility and showing promising results in the accuracy of brain tumor detection.

## Materials and methods

The machine-learning model used in this study was trained using the "Brain Tumor MRI Dataset," an open-source online database of brain MRI images curated by Msoud Nickparvar via Kaggle [17]. This dataset amalgamates three distinct datasets: figshare, SARTAJ, and BR35H, comprising a total of 7,023 images. For the purposes of this research, a subset of 2,000 images was selected, encompassing brain MRIs of gliomas (500 images), meningiomas (500 images), pituitary tumors (500 images), and normal scans without tumors (500 images).

The image classification model was constructed using Google Teachable Machine (Alphabet Inc., Mountain View, CA), a widely used online platform facilitating the testing, training, and deployment of machine learning classification models [18]. The training process encompassed 80 epochs with a batch size of 32 and a learning rate set at 0.0001. Each image was adjusted to ensure a 1:1 aspect ratio. The primary objective during training was to evaluate Google Teachable Machine's (GTM) efficacy in discerning between gliomas, meningiomas, and normal scans devoid of tumors.

An epoch, indicating the number of times each image traverses the training model, and batch size, denoting the number of images used per training iteration, were carefully considered. With a total of 63 batches given 2,000 images and a batch size of 32, each epoch concludes once all batches have been processed.

GTM autonomously partitions its dataset into training and test samples, allocating 85% of the images for training (425 images per class) and 15% for testing (75 images per class), a split unmodifiable by the user. While GTM provides a confusion matrix and accuracy metrics, additional parameters such as precision, recall, and F1 score were derived through statistical analyses using the generated plots and accuracy data to enhance the quantification of GTM's performance in distinguishing between various types of brain scans with tumors.

## Results

The performance of the AI model in classifying brain images across different tumor categories was evaluated using several metrics, as shown in Table 1. The table summarizes the performance metrics of a classification model used to classify four different classes: "normal" (no tumor), "glioma," "meningioma," and "pituitary." Each class is evaluated based on four metrics: accuracy, precision, recall, and F1 score. For normal cases, the model exhibited an accuracy and precision of 0.96, a recall of 0.92, and an F1 score of 0.94, indicating high accuracy and precision with a few false negatives. In the case of glioma, the model demonstrated perfect accuracy and precision (1.00), with a slightly lower recall of 0.94 and an F1 score of 0.97, suggesting a few false negatives. For meningioma cases, the model had an accuracy and precision of 0.84, a high recall of 0.97, and an F1 score of 0.90, implying good identification of true positives but a higher rate of false positives. For pituitary cases, the model performed highly with an accuracy and precision of 0.97, a recall of 0.94, and an F1 score of 0.95. In the evaluation of the diagnostic model, the rates of false negatives were observed to be 8% for the normal class, 6% for both the glioma and pituitary classes, and 3% for the meningioma class. Furthermore, the analysis of false positive rates, derived from the precision values, revealed that the normal and pituitary classes had a rate of 4% and 3% respectively, while the glioma class demonstrated no false positives. However, the meningioma class exhibited a higher false positive rate of 16%. Overall, the model performs well across all classes, with the highest performance observed in the glioma class.

**Table 1:** Performance metrics of the classification model used to classify four different classes: normal, glioma, meningioma, and pituitary. Each class is evaluated based on four metrics: accuracy, precision, recall, and F1 score.

Class	Accuracy	Precision	Recall	F1 score
Normal	0.96	0.96	0.92	0.94
Glioma	1	1	0.94	0.97
Meningioma	0.84	0.84	0.97	0.9
Pituitary	0.97	0.97	0.94	0.95

These metrics provide insights into the model's performance. For instance, an accuracy of 0.96 for the "no tumor" class implies that 96% of the images without tumors were correctly classified. A precision score of 0.96 for the "no tumor" class indicates that 96% of the images predicted as "no tumor" were indeed without tumors. A recall of 0.92 for the "no tumor" class suggests that 92% of the actual images without tumors were correctly identified. An F1 score of 0.94 for the "no tumor" class reflects a strong balance between precision and recall for this category.

**Figure 1:**
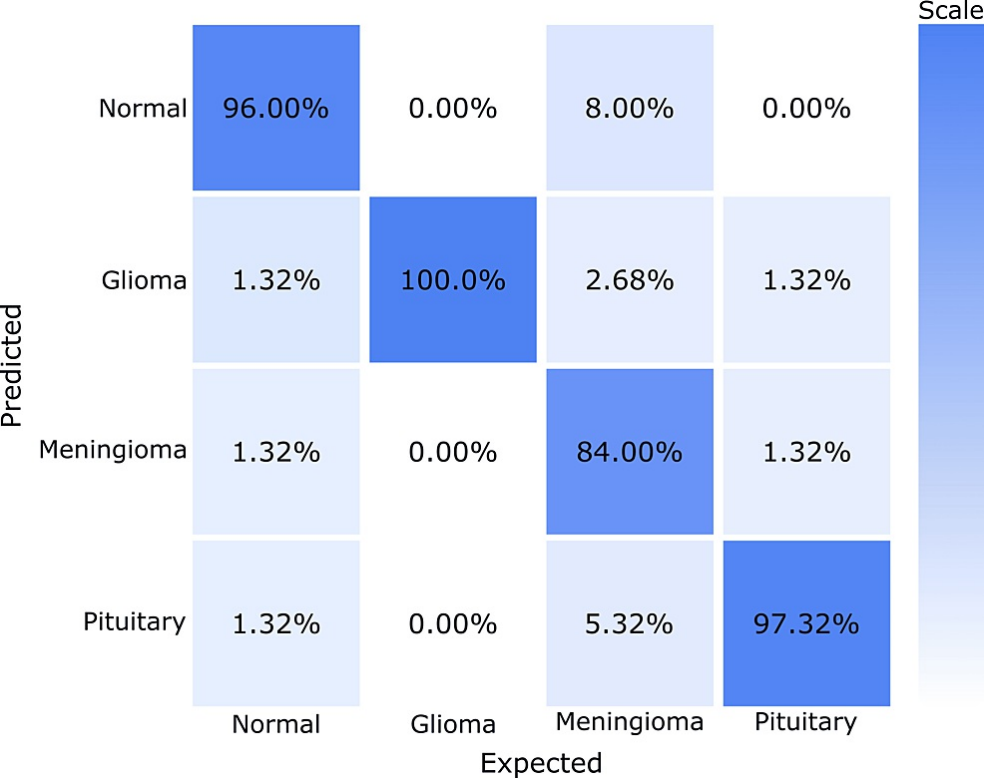
A 4x4 confusion matrix showing classification accuracy for four different conditions with true positive rates as follows: 'normal' at 96% accuracy, 'glioma' at 100% accuracy, 'meningioma' at 84% accuracy, and 'pituitary' at 97% accuracy, with low misclassification between conditions.

The confusion matrix data (Figure 1) further confirms these metrics. The off-diagonal values, ranging from 0 to 8%, suggest some misclassifications occur, but overall, error rates are low. This suggests the model is highly capable of correct diagnoses with a low likelihood of mistaking one condition for another.

**Figure 2:**
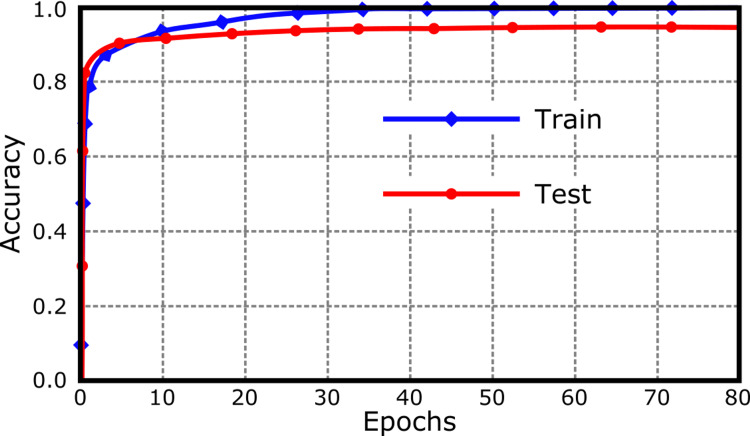
The GTM-generated accuracy plot shows rapid learning with the training accuracy quickly reaching perfect scores by the 30th epoch. The test accuracy also climbs swiftly, indicating good model generalization, and stabilizes at a high value just above 0.9, suggesting robust predictive performance on unseen data.

The accuracy plot generated from GTM, shown in Figure 2, reveals a good level of model performance. The training curve shows a rapid ascent to high accuracy, hitting the 0.8 mark right at the outset and achieving perfection with a 1.0 accuracy from the 30th epoch onwards. The test accuracy curve also starts strong, similarly breaching the 0.8 threshold early in training, yet rather than reaching perfection, it levels off to a value just above 0.9. This still represents a very high accuracy and indicates that the model generalizes well to unseen data, albeit with a slight performance drop compared to the training data.

**Figure 3:**
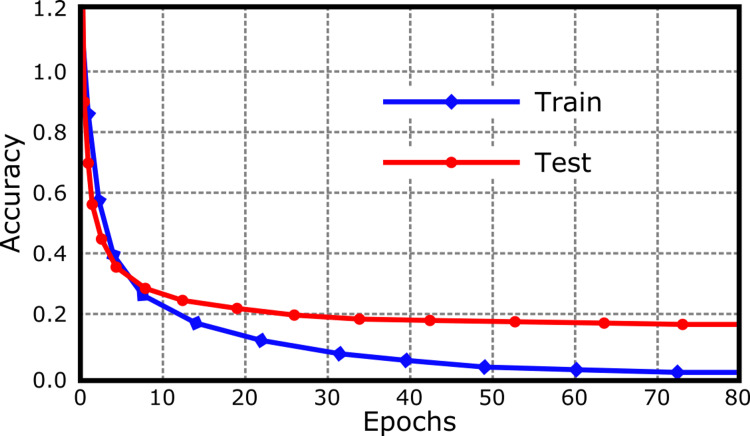
The GTM loss plot displays a learning progression with the training loss decreasing from an initial value above 1.0 to 0.0 by 80 epochs, illustrating the model's improving accuracy on training data. The test loss follows a similar decline but levels off just under 0.2, indicating a good generalization to unseen data with a small, stable margin of error.

The loss plot generated from GTM, shown in Figure 3, illustrates the model's learning process over time. The training curve beginning above 1.0 and steadily decreasing to 0.0 by the 80th epoch signifies that the model is becoming increasingly effective at predicting the training data with fewer errors. The test curve's quick decline mirrors the training curve but leveling off just under 0.2 suggests that while the model reduces loss significantly for the test data, it does not reach the same level of minimal loss as with the training data.

Despite the plateau, a test loss value of just under 0.2 is relatively low and indicates that the model has a strong predictive capacity on unseen data. It suggests that while the model may be fine-tuned to the training examples, it is likely to perform well in practice, potentially assisting clinicians in making informed decisions based on its predictions. However, it is crucial to validate these observations with additional real-world data to ensure the model's practical applicability and to confirm that it can maintain a low error rate in diverse clinical scenarios.

## Discussion

Our group has a history of exploring the applications of machine learning in various healthcare and medical contexts, ranging from patient safety enhancement through integrated sensor technology [19], to the prediction of coronary artery disease [20], cardiovascular health management in diabetic patients [21], and image detection of colonic polyps [22]. We have also contributed to the academic discourse on the transformative potential of AI in healthcare, navigating the ethical landscape, and public perspectives through our review paper [23]. Furthermore, our entry paper on predictive modeling in medicine underscores our commitment to harnessing the power of machine learning for improved healthcare outcomes [24]. The current study, focusing on the classification of brain tumors using a machine learning model built on the GTM platform, is a natural extension of our previous work. It leverages the advancements in machine learning, particularly in the realm of medical imaging, to develop a model that can accurately identify different types of brain tumors. The promising results of this study, coupled with our previous research, underscore the potential of machine learning as a powerful tool in healthcare, capable of enhancing diagnostic accuracy and ultimately improving patient outcomes. 

The primary objective of this investigation was to develop an image classification model using machine learning methodologies, specifically applied to a comprehensive brain MRI dataset. The model was trained using GTM's standard image classification model, which facilitated the training process. The outcomes of the image classification model showcased promising levels of accuracy, precision, recall, and F1 score. The "no tumor" class achieved an accuracy of 0.96, indicating the correct classification of 96% of tumor-free images by the model. The glioma class achieved a perfect accuracy score of 1.00, affirming the model's adept identification of all glioma images. In comparison, the meningioma class achieved an accuracy of 0.84, while the pituitary tumor class achieved an accuracy of 0.97. The mean accuracy across all classes was 0.94, underscoring the model's overall efficacy.

Observations from accuracy and loss plots revealed a positive correlation between increased epochs and enhanced training and test accuracy. However, with prolonged epochs, a divergence in loss between the training and test splits emerged, indicating potential overfitting. This phenomenon suggests the model's excessive specialization on training data, compromising its adaptability to unseen data.

The model presented has a few drawbacks. Its capacity to generalize across different patient populations or imaging methods may be limited or biased due to its reliance on a single dataset, no matter how large. The robustness of the model and its application to actual clinical settings are hampered by the lack of external validation datasets. To improve the model's capacity for generalization, more varied datasets should be included. The observed overfitting tendencies highlight the necessity for additional research into regularization strategies. Even though the model showed encouraging performance measures, more validation in larger cohorts and real-world settings is needed to determine the model's dependability and effectiveness in realistic tumor classification tasks before it can be applied clinically.

These findings underscore the effectiveness of the AI model in accurately categorizing brain images across various tumor types. Despite its remarkable precision and accuracy, particularly evident in the glioma class, the model displayed comparatively diminished recall in the meningioma class. While F1 scores highlighted a harmonious balance between precision and recall across most tumor categories, concerns regarding potential overfitting surfaced from observed loss plots. 

Addressing this challenge warrants further investigation and the adoption of techniques like regularization or early stopping to enhance model robustness. Regularization techniques involve shrinking model coefficients to minimize loss, thus curbing overfitting without compromising training accuracy [25,26]. Additionally, augmenting training data with diverse edge cases could help mitigate overfitting and bolster the model's generalization capabilities. Continued research and refinement efforts are imperative to mitigate overfitting concerns and elevate the model's overall performance and reliability in clinical tumor classification applications.

The accurate distinction of tumor pathologies from non-tumor mimickers such as bacterial abscesses, toxoplasma, and tuberculomas is of paramount importance. Misinterpretations can lead to erroneous diagnoses, such as mistaking an abscess for a glioma, which can significantly impact the treatment plan and prognosis. This underscores the need for advanced diagnostic tools that can accurately differentiate between these conditions. Machine learning models, trained on comprehensive datasets encompassing a wide range of pathologies, could potentially address this challenge. These models could learn the subtle differences in imaging characteristics between tumor and non-tumor conditions, thereby enhancing diagnostic accuracy. However, the effectiveness of such models would be contingent on the quality and diversity of the training data, necessitating the inclusion of a broad spectrum of both tumor and non-tumor pathologies. Future research should focus on developing and validating such models, with the ultimate aim of aiding radiologists in making more accurate diagnoses.

## Conclusions

This study developed and evaluated an image classification model using machine learning techniques applied to brain MRI datasets. The model demonstrated promising accuracy, precision, recall, and F1 scores across various tumor categories, indicating its potential utility in clinical settings for tumor classification tasks. However, the study also identified inherent limitations in the model, including dataset bias, overfitting tendencies, and the absence of external validation datasets. These limitations highlight the need for further refinement and validation efforts. To address these issues, future work should focus on incorporating diverse datasets, implementing regularization techniques, and validating the model in larger cohorts and real-world scenarios. These steps will be crucial for enhancing the model's reliability and applicability in clinical practice.

Despite these challenges, the findings of this study lay a solid foundation for continued advancements in AI-driven diagnostic tools for brain tumor classification. This work ultimately contributes to improved patient care and outcomes in neuroimaging, paving the way for future research and development in this critical area of healthcare. This study underscores the potential of machine learning in enhancing diagnostic accuracy and efficiency in neuroimaging, and it serves as a stepping stone towards the broader application of AI in healthcare.
